# Safety and efficacy of Chinese herbal bath for psoriasis vulgaris: A protocol for systematic review and meta-analysis

**DOI:** 10.1097/MD.0000000000020488

**Published:** 2020-05-22

**Authors:** Wenxia Lin, Qianying Yu, Yuesi Qin, Jianhua Xiao, Li Peng, Jie Zhang, Jia He, Haoyu Xiang, Min Xiao, Mingling Chen

**Affiliations:** a Department of Dermatology, Hospital of Chengdu University of Traditional Chinese Medicine; b School of Clinical Medicine, Chengdu University of Traditional Chinese Medicine; c Department of Dermatology, Chengdu Yinkang Psoriasis Hospital, Chengdu, Sichuan Province, P.R. China.

**Keywords:** Chinese herbal bath, protocol, psoriasis vulgaris, randomized controlled trials, systematic review

## Abstract

**Background:**

Psoriasis is a common, chronic, and recurrent skin inflammatory disease, with psoriasis vulgaris considered as the most prevalent type of psoriasis. Chinese herbal bath, a type of traditional Chinese medicine, is an external therapy widely used to treat psoriasis vulgaris in China, and it has achieved satisfactory clinical effects. However, there are few studies evaluating the safety and efficacy of Chinese herbal bath compared with other external therapies administered under similar conditions. The purpose of this study is to comprehensively evaluate the clinical safety and efficacy of Chinese herbal bath in the treatment of psoriasis vulgaris through a systematic evaluation of the literature, so as to provide a reference basis for future clinical applications.

**Methods:**

PubMed, Embase, CENTRAL, the Web of Science, the China Biology Medicine Database (CBM), the China National Knowledge Database (CNKI), the Wan Fang Database, and the Chong Qing VIP Database will be searched to collect randomized controlled trials of Chinese herbal bath used to treat psoriasis vulgaris. The search time limits will be from the establishment of the database to December 2019. Two researchers will independently screen the studies, extract data, and evaluate the risk of bias of the studies. Meta-analysis will be carried out with the RevMan5.3 software. The mean difference will be used as the effect index for the measurement data, and the odds ratio will be used as the effect index for the enumeration data. The 95% confidence interval will be provided for each effect. Heterogeneity among the results of each study will be evaluated by the Chi-square test.

**Results:**

This study will comprehensively evaluate the clinical safety and efficacy of Chinese herbal bath in the treatment of psoriasis vulgaris, so as to provide a reference basis for future clinical applications.

**Conclusion:**

This study will provide a theoretical basis for the standardized administration of Chinese herbal bath.

**OSF registration number:**

doi: 10.17605/OSF.IO/4HRPJ

## 1 Introduction

Psoriasis is a common, chronic, recurrent, and inflammatory skin disease that is classified into four subtypes: common, articular, pustular, and erythrodermic. The global incidence rate is ∼2%. Psoriasis vulgaris is the most common type, accounting for ∼80% to 90% of psoriasis patients.^[1–3]^ Patients with psoriasis not only experience skin pain, pruritus, and other discomforts, but also carry serious psychological burdens, including social embarrassment, anxiety, and depression, which can affect their quality of life.^[4,5]^ In addition, patients with psoriasis are more likely to suffer from other chronic diseases. These comorbidities include psoriatic arthritis, Crohn's disease, cancer, depression, nonalcoholic fatty liver disease, cardiovascular disease, and various metabolic syndromes, all of which greatly contribute to the morbidity and mortality of patients with psoriasis.^[2]^ Specifically, severe psoriasis is associated with an increased risk of death from a variety of causes, with central vascular death considered as the most common cause.^[6]^ For a long time, psoriasis was defined as a multi-system inflammatory disease mainly affecting the skin. Although psoriasis can impact the quality of life, challenges remain in the diagnosis and treatment of this prevalent disease.^[7]^ Furthermore, advancements in the treatment of psoriasis have been slow. However, there are many ways to alleviate or halt symptoms with immunomodulatory, anti-inflammatory, phototherapeutic, and biological agents either alone or in combination with alternative or sequential therapies. Although the clinical effects are generally positive, different degrees of skin irritation and adverse reactions may occur, especially with long-term use of oral drugs, which can increase medical costs and reduce the quality of life.^[8,9]^

Currently, the most effective drugs are biological agents, but they are too expensive for long-term treatment.^[10]^ Compared with chemical and biological therapies, Chinese herbal bath is cost-effective, relatively safe with few adverse reactions, and effective for the treatment of mild psoriasis. Chinese herbal bath is widely used in the treatment of psoriasis because it can improve external skin lesions and regulate immune responses.^[9,11,12]^ Several systematic reviews have reported that Chinese herbal bath combined with other therapies show good effects in the treatment of psoriasis vulgaris.^[13–15]^

Chinese herbal bath, as an external therapy, can alleviate disease symptoms. It involves adding a specific quantity of Chinese herbal medicine to bathwater, followed by heating the water to a suitable temperature and bathing the whole body or part of the body for a specific time period.^[16]^ Because of its effectiveness, simplicity, convenience, and cost-effectiveness, Chinese herbal bath has recently gained popularity.

The principle of percutaneous absorption contends that Chinese herbal bath enhances contact between the lesion and the components of Chinese herbal medicine, thereby promoting percutaneous absorption of traditional Chinese medicine. Chinese herbal bath also does not induce a first-pass effect on the digestive tract. The principle of reflexive regulation of the nervous system contends that Chinese herbal bath regulates vasomotion and acupoint stimulation, whereas the principle of hydration contends that Chinese herbal bath increases the penetration and absorption of volatile substances, polysaccharides, vitamins, and other components in Chinese herbal medicines.^[17]^ The clinical use of Chinese herbal bath either alone or in combination with other therapies to treat psoriasis vulgaris has recently gained popularity. Compared with other external therapies administered under similar conditions, there are few studies evaluating the safety and efficacy of Chinese herbal bath in the treatment of psoriasis vulgaris. Thus, the purpose of this study is to comprehensively evaluate the clinical safety and efficacy of Chinese herbal bath in the treatment of psoriasis vulgaris through a systematic evaluation of the literature, so as to provide a reference basis for future clinical applications.

## 2 Methods

This protocol has been registered with the Open Science Framework (OSF) (registration number: DOI 10.17605/OSF.IO/4HRPJ). The review of this system will be submitted to peer-reviewed journals. This protocol was reported according to preferred reporting items for systematic reviews and meta-analysis protocols (PRISMA-P) 2015 statement.^[18]^

### 2.1 Inclusion criteria

#### 2.1.1 Type of study

All randomized controlled trials (RCTs) that evaluate the clinical safety and efficacy of Chinese herbal bath in the treatment of psoriasis vulgaris will be included.

#### 2.1.2 Types of participants

Patients diagnosed with psoriasis vulgaris will be 18 to 65 years old, regardless of gender and course of the disease.

#### 2.1.3 Types of interventions

The experimental group will be given Chinese herbal bath, whereas the control group will be given another external therapy. The two groups will follow all other basic treatments as instructed.

### 2.2 Types of outcome measures

#### 2.2.1 Primary outcomes

PASI^[19]^ scores will be evaluated as the primary outcome. The psoriasis area and severity index is a reliable, reproducible and responsive instrument. It has the advantages of internal consistency, intra-observer reliability, and inter-observer reliability. It is considered as gold standard for assessing the severity of cutaneous manifestations in psoriasis. Therefore, it is the preferred outcomes.^[20]^

#### 2.2.2 Secondary outcomes

When treating psoriasis with Traditional Chinese Medicine in China, the evaluation standard of Traditional Chinese Medicine is usually adopted, and the Guiding Principles for Clinical Research of New Traditional Chinese medicine (trial) is the general evaluation standard in China. According to the evaluation standard, the effective rate of treatment, pruritus score, and incidence of adverse reactions are taken as secondary outcomes.^[21]^

### 2.3 Exclusion criteria

The exclusion criteria will be as follows: patients who do not meet the inclusion criteria; patients allergic to drugs or drug components; patients who have taken steroids within the preceding 2 weeks and/or taken retinoids or topical steroids within the preceding week; patients with active pulmonary tuberculosis, acute or chronic hepatitis, and other infectious diseases; pregnant or lactating women; studies involving animal experiments; duplicate studies; and studies with no outcomes.

### 2.4 Electronics searches

PubMed, Embase, CENTRAL, the Web of Science, the China Biology Medicine Database (CBM), the China National Knowledge Database (CNKI), the Wan Fang Database, and the Chong Qing VIP Database will be searched to collect RCTs of Chinese herbal baths used to treat psoriasis vulgaris. The search time limit will be from the establishment of the database to December 2019. In addition, other literature resources will be thoroughly searched to identify all articles meeting the requirements. These resources will include the Chinese Clinical Trial Registry (http://www.chictr.org.cn), conference papers, Master and Doctorate degree studies.

### 2.5 Search strategy

The searches will be carried out using a combination of subject headings and free words. Keywords will include “drugs, Chinese herbal,” “Chinese drugs, plant”, “Chinese herbal drugs,” “herbal drugs, Chinese,” “plant extracts, Chinese,” “Chinese plant extracts,” “extracts, Chinese plant,” “balneology,” “balneotherapy,” “hydrotherapy,” “psoriasis,” “psoriases,” “psoriasis vulgaris,” “plaque psoriasis,” “guttate psoriasis,” and “randomized controlled trial.”

### 2.6 Data extraction and management

**Figure 1:**
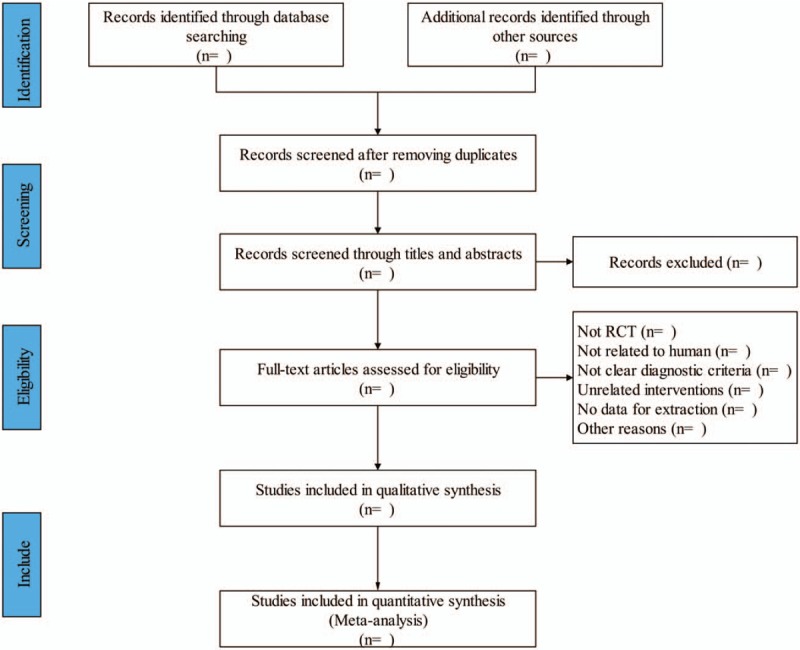
Flow diagram of the study selection process.

Two researchers (Wenxia Lin and Yuesi Qin) will independently screen the studies, extract data, and perform crosschecks. Differences will be solved through discussion with a third researcher (Mingling Chen). The article screening process will involve reading the title first, followed by the abstract and full text to determine whether the study should be included. The process of study selection is shown in flow diagram (Fig. 1).

The following information will be extracted from the studies: basic information, including the research topic, first author, and publication time; sample size, intervention measures, and treatment course; key elements of bias risk assessment; and outcome indicators and outcome measurement data.

### 2.7 Assessment of risk of bias

Two researchers (Wenxia Lin and Yuesi Qin) will independently evaluate the risk of bias according to the bias risk assessment tool for RCTs as given in the Cochrane manual.^[22]^ The bias risk assessment categories will include the following parameters: random sequence generation, allocation concealment, blinding of participants and personnel, blinding of outcome assessment, incomplete outcome data, selective reporting, and other biases. Each bias risk assessment will be classified into one of three levels: low risk, unknown risk, or high risk.

### 2.8 Data synthesis and analysis

Meta-analysis will be performed with RevMan V.5.3 software. SMD will be used for continuous variables, and the OR will be used for dichotomous data. The 95% CI will be given for each effect size.

#### 2.8.1 Assessment of heterogeneity

The Chi-square test will be used to evaluate the statistical heterogeneity of the results of each study (*α* = 0.1), and *I*^2^ will be used to quantitatively determine the heterogeneity. If there is no statistical heterogeneity (*I*^2^ < 50% and *P* > .1), the fixed-effects model will be used for meta-analysis. If there is statistical heterogeneity (*I*^2^ > 50% and *P* ≤ .1), the source of heterogeneity will be further analyzed, and the random-effects model will be used for meta-analysis after the effect of the obvious clinical heterogeneity is excluded. The meta-analysis of coefficient *α* will be set at 0.05. *P* < .05 will be considered as statistically significant. The obvious clinical heterogeneity will be treated by subgroup analysis, sensitivity analysis, or descriptive analysis.

#### 2.8.2 Subgroup analysis

If there is significant heterogeneity in the included studies, we will conduct subgroup analysis according to the bath time, treatment course, or treatment frequency.

#### 2.8.3 Sensitivity analysis

Sensitivity analysis will be used to evaluate whether the results of the meta-analysis are stable and reliable. The weak studies will be removed from the robust studies, and meta-analysis will be conducted again to determine whether there is a significant difference in the effect size before and after the removal of the weak studies. If there is no significant change after the removal of the weak studies, then the sensitivity is low, and the results will be credible. In contrast, if there is a significant change or the findings are opposite after the removal of the weak studies, then the sensitivity is high, and the results will be less credible. If quantitative synthesis is not appropriate, a comprehensive narrative will be provided through the text and tabular information to summarize and explain the characteristics and findings of the literature, and to explore the relationship within or between the literatures.

#### 2.8.4 Assessment of publication bias

If ≥10 studies, funnel plots will be used to assess publication bias. A symmetrical distribution of funnel plot data shows that there is no publication bias. If there is significant publication bias, then asymmetric funnel plots will be generated. More obvious asymmetries will indicate higher degrees of bias.

#### 2.8.5 Evidence quality evaluation

According to the quality evaluation standard of GRADE,^[23]^ the quality of evidence will be classified into four grades: high quality, moderate quality, low quality, and very low quality. A grade of high quality will indicate that further research is very unlikely to change our confidence in the effect estimate. A grade of moderate quality will indicate further research may have a significant impact on our confidence in the effect estimate and may change the estimate. A grade of low quality will indicate that further research is likely to have a significant impact on our confidence in the effect estimate and is likely to change the estimate. A grade of very low quality will indicate that any estimate of the effect is highly uncertain.

## 3 Discussion

In recent years, with the gradual understanding of the pathogenesis of psoriasis and the continuous improvement of medical level, psoriasis has made great progress in treatment measures, but it is still in the stage of disease control and can not be completely cured. Long-term treatment, heavy economic burden and easy recurrence have become the main factors affecting the quality of life of patients with psoriasis. Chinese herbal bath is an external treatment method to alleviate the disease by adding a certain amount of Chinese herbs to the water and bathing the whole body or part of the body at an appropriate temperature.^[16]^ Chinese herbal bath directly acts on the affected skin, effectively improves local blood circulation, promotes skin damage repair, and is increasingly used in the treatment of psoriasis vulgaris. Although there was a systematic reviews has reported that Chinese herbal bath combined with other therapies show good effects and safety in the treatment of psoriasis vulgaris,^[14]^ however, our research is quite different from theirs. First, there are different interventions. In their study, the intervention levels of the observation group and the control group are different, and the observation group had more interventions than the control group. Our research will be based on the same levels of intervention measures between the observation group and the control group, which makes the Chinese herb bath comparable with other therapies, so as to more objectively evaluate the clinical efficacy and safety of Chinese herb bath. Secondly, there are different diagnostic criteria. In their study, the diagnostic criteria are not unified, all the 14 literatures included, only 3 literatures met the inclusion criteria. Our study will ensure the unity of inclusion criteria and reduce the risk of bias between inclusion literatures. Although both of our studies are to evaluate the clinical efficacy and safety of Chinese herb bath in the treatment of psoriasis vulgaris, in view of the differences between the two, our research will be more objective and the results will be more valuable.

## Author contributions

**Conceptualization:** Wenxia Lin, Mingling Chen.

**Investigation:** Qianying Yu, Yuesi Qin.

**Supervision:** Min Xiao, Jianhua Xiao.

**Writing – original draft:** Jia He, Haoyu Xiang.

**Writing – review & editing:** Li Peng, Jie Zhang.
